# Establishment of a mouse model for the complete mosquito-mediated transmission cycle of Zika virus

**DOI:** 10.1371/journal.pntd.0006417

**Published:** 2018-04-18

**Authors:** Yi-Ping Kuo, Kuen-Nan Tsai, Yin-Chiu Luo, Pei-Jung Chung, Yu-Wen Su, Yu Teng, Ming-Sian Wu, Yu-Feng Lin, Chao-Yang Lai, Tsung-Hsien Chuang, Shih-Syong Dai, Fan-Chen Tseng, Cheng-Han Hsieh, De-Jiun Tsai, Wan-Ting Tsai, Chun-Hong Chen, Guann-Yi Yu

**Affiliations:** 1 National Institute of Infectious Diseases and Vaccinology, National Health Research Institutes, Zhunan, Taiwan; 2 Institute of Molecular and Genomic Medicine, National Health Research Institutes, Zhunan, Taiwan; 3 National Mosquito-Borne Diseases Control Research Center, National Health Research Institutes, Zhunan, Taiwan; 4 Immunology Research Center, National Health Research Institutes, Zhunan, Taiwan; 5 College of Nursing, National Taipei University of Nursing and Health Sciences, Taipei, Taiwan; University of Florida, UNITED STATES

## Abstract

Zika virus (ZIKV) is primarily transmitted by *Aedes* mosquitoes in the subgenus *Stegomyia* but can also be transmitted sexually and vertically in humans. STAT1 is an important downstream factor that mediates type I and II interferon signaling. In the current study, we showed that mice with STAT1 knockout (*Stat1*^-/-^) were highly susceptible to ZIKV infection. As low as 5 plaque-forming units of ZIKV could cause viremia and death in *Stat1*^-/-^ mice. ZIKV replication was initially detected in the spleen but subsequently spread to the brain with concomitant reduction of the virus in the spleen in the infected mice. Furthermore, ZIKV could be transmitted from mosquitoes to *Stat1*^*-/-*^ mice back to mosquitoes and then to naïve *Stat1*^*-/-*^ mice. The 50% mosquito infectious dose of viremic *Stat1*^*-/-*^ mouse blood was close to 810 focus-forming units (ffu)/ml. Our further studies indicated that the activation of macrophages and conventional dendritic cells were likely critical for the resolution of ZIKV infection. The newly developed mouse and mosquito transmission models for ZIKV infection will be useful for the evaluation of antiviral drugs targeting the virus, vector, and host.

## Author summary

Zika virus (ZIKV) is transmitted mainly by mosquito bites and can also be transmitted between humans by sex or from pregnant women to their babies. ZIKV infection causes damage in many tissues including the brain in adults and newborns, making ZIKV infection an important health issue globally. To develop new tools for ZIKV research, we determined that a genetically modified mouse strain, *Stat1*^-/-^, was highly sensitive to ZIKV infection. We also demonstrated that ZIKV could be delivered to mice by mosquito bites and transmitted back to *Stat1*^-/-^ mice. The newly developed mouse model will be useful for developing new strategies to treat ZIKV infection and for studying mechanisms to reduce mosquito-mediated transmission.

## Introduction

Zika virus (ZIKV) is an enveloped, positive sense RNA virus belonging to the Flaviviridae family and is primarily transmitted to humans via the bite of an *Aedes aegypti* mosquito [1, 2]. ZIKV infections cause self-limiting fever, headache, myalgia, and conjunctivitis [1]. A recent large ZIKV outbreak in Brazil in 2015–2016 was linked to an increased incidence of microcephaly and congenital malformations in children born in the epidemic area as a result of ZIKV crossing the placenta of infected mothers [3–6]. ZIKV infection was linked to Guillain-Barré syndrome in adults [7, 8]. After an acute infection phase, ZIKV can establish a persistent infection in the male reproductive tract that can then be sexually transmitted [9, 10]. These varied transmission routes and different clinical presentations make ZIKV a serious public health threat.

Due to the complexity of the ZIKV transmission cycle, which involves a vertebrate host and a mosquito vector, an animal model paralleling the infection process in humans would be useful to better understand the disease process resulting from ZIKV infections as well as in the evaluation of antiviral compounds and vaccine candidates. Because mice are naturally immune to a ZIKV infection, mouse strains defective in antiviral immune pathways such as AG129, *Ifnar*^*-/-*^, *Stat1*^-/-^, *Stat2*^-/-^ and *Irf3*^*-/-*^*Irf5*^*-/-*^*Irf7*^*-/-*^ triple knock out mice have been used in ZIKV pathogenesis research [11–15]. For instance, ZIKV-infected *Ifnar*^*-/-*^ mice developed weight loss, paralysis, and a systemic virus infection associated with an age-dependent mortality rate (20–100%), that is, young mice (3–4 weeks old) were more sensitive to ZIKV infection than aged mice (11–12 weeks) [11, 12]. ZIKV caused a systemic infection detectable in several organs of these immunocompromised animals including the spleen, kidney, testes, and brain [11, 12]. Immune-deficient mice also have been used to investigate the competence of *Aedes* mosquitoes to various ZIKV strains [2, 16]. Interestingly, blood meals from ZIKV viremic mice are more infectious to mosquitoes than artificial blood meals of comparable doses [2]. The relative contributions of various factors from host and vector to ZIKV transmission remain to be explored.

The contribution of the mosquito bite on the host's inflammatory response and how this impacts virus dissemination *in vivo* has been shown to play a role in infection models of Semliki Forest virus and Bunyamwera virus [15]. The role that the mosquito plays in ZIKV pathogenesis is undefined due to few infection models that involve the transmission of the virus via a mosquito [17]. STAT1 and STAT2 are key transcription factors activated by type I IFN signaling that are induced following infections with various pathogens including viruses [18]. ZIKV antagonizes type I IFN response through the suppression of STAT1 phosphorylation and STAT2 degradation [19–21]. *Stat1*^*-/-*^ mice was recently utilized in anti-ZIKV drug development [15]. In the presented study, we found that ZIKV caused systemic infections in *Stat1*^*-/-*^ mice that presented with high viremia and brain infections. In addition, we demonstrate that ZIKV can directly be transmitted between *A*. *aegypti* mosquitoes and *Stat1*^*-/-*^ mice. The mosquito-dependent ZIKV transmission mouse model will be useful in studies of disease progression in the testing of novel antiviral therapies and anti-transmission strategy.

## Results

### ZIKV replicates in *Stat1*^-/-^ mice

**Fig 1:**
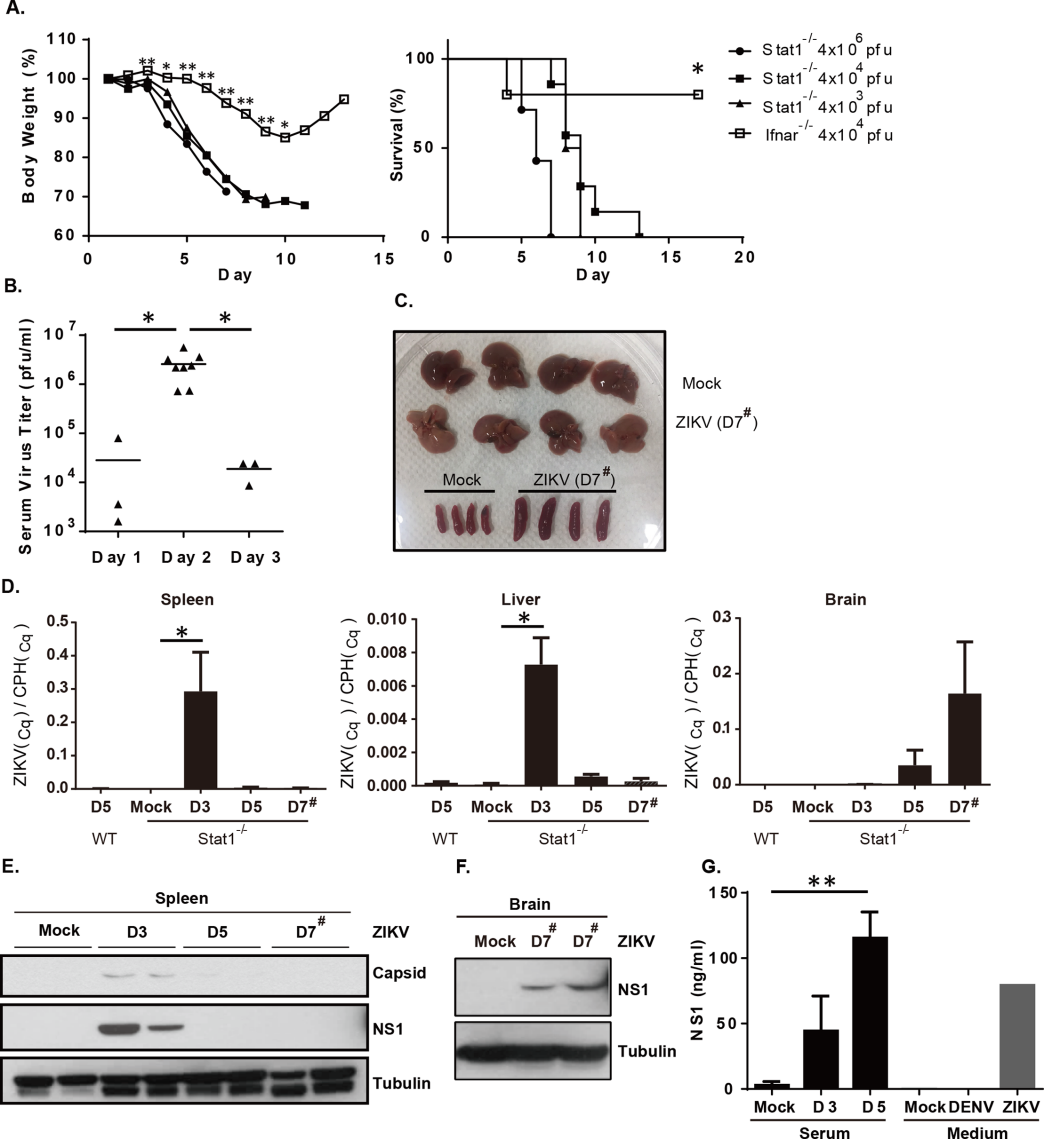
Infection of *Stat1*^-/-^ mice with ZIKV. **(A)** 12–16 week-old *Stat1*^-/-^ mice (n = 4–7) were infected intraperitoneally (i.p.) with various doses of ZIKV (4×10^3^, 4×10^4^, or 4×10^6^ pfu/mouse). Weights and survival rates were monitored daily. Nine week-old *Ifnar*^-/-^ mice were infected i.p. with 4×10^4^ pfu/mouse in parallel. **(B)** Serum samples were collected from ZIKV infected *Stat1*^-/-^ mice (4×10^4^ pfu/mouse) and virus titers were measured by plaque assay. **(C)** Photograph of liver and spleen collected from ZIKV infected (1×10^3^ pfu/mouse) (D7^#^) and uninfected *Stat1*^-/-^ mice (Mock) on D7 post-infection. **(D)** ZIKV and cyclophilin A (CPH, a house keeping gene) RNA expression in various tissues was measured by quantitative RT-PCR (n = 3–4) to obtain quantification cycle (Cq) values. Relative ZIKV gene expression level was calculated as ZIKV(_Cq_)/CPH(_Cq_). D3 and D5 samples were collected from mice infected with 4×10^4^ pfu/mouse and D7^#^ samples were from mice infected with 1×10^3^ pfu/mouse. **(E)** ZIKV viral protein expression in spleens harvested at D3 and D5 post-infection (4×10^4^ pfu/mouse) and D7 post-infection (1×10^3^ pfu/mouse) were examined by immunoblotting with specific antibodies for NS1 and capsid. **(F)** NS1 protein expression in spleens and brains 7 days post-infection (1×10^3^ pfu/mouse) was also evaluated. **(G)** Determination of serum NS1 concentrations by ELISA. Culture medium collected from Vero cells infected with DENV or ZIKV were used as a positive control for NS1. *, p ≤ 0.05; **, p ≤ 0.01.

STAT1 is a key transcription factor activated following signals delivered by type I and II interferons that result in the activation of antiviral-related genes [18]. To test the sensitivity of *Stat1*^-/-^ mice to ZIKV infection, adult *Stat1*^-/-^ mice were intraperitoneally challenged with 4x10^3^ to 4x10^6^ ZIKV plaque forming units (pfu)/mouse that resulted in significant weight loss and 100% mortality by two weeks at the doses tested (Fig 1A). In contrast, ZIKV-infected *Ifnar*^-/-^ mice showed much less weight loss and only had a 20% mortality rate (p = 0.0199) when challenged with 4x10^4^ pfu/mouse compared to the *Stat1*^-/-^ mice with the same ZIKV infection dose. It has been shown that young *Ifnar*^-/-^ mice were more sensitive to ZIKV infection compared to old mice [12]. The age of *Ifnar*^-/-^ mice used in Fig 1A was 9 weeks old and *Stat1*^-/-^ mice were 12–16 weeks old. Age or gender differences did not affect the mortality rates observed in *Stat1*^-/-^ mice. In the ZIKV-infected *Stat1*^-/-^ mice (4×10^4^ pfu/mouse), viremia peaked at 2 days post-infection (Fig 1B, p = 0.0095), and declined in blood over time. ZIKV-infected mice also presented with splenomegaly and were moribund. Some of the infected mice had pale livers (Fig 1C). Taken together, *Stat1*^-/-^ mice were more sensitive to ZIKV infection than *Ifnar*^-/-^.

### ZIKV replication occurs initially in the spleen and later in the brain

To examine the kinetics of ZIKV replication in *Stat1*^-/-^ mice, viral RNA was isolated from various organs including liver, spleen, and brain and analyzed by quantitative real-time PCR (RT-PCR). ZIKV RNA was detected in peripheral organs, spleen and liver, at early time points following infection and by day 5 began to decline (Fig 1D). Because mice infected with 4x10^4^ pfu were dead or moribund by day 7 post-infection, the low infection dose (1x10^3^ pfu/mouse) was used during the examination of tissues harvested at this time point (Fig 1D). High levels of ZIKV RNA were detected in spleens 3 days post-infection suggesting that this organ could be the main replication site initially prior to systemic dissemination. In contrast, viral RNA in brain tissues was not detected until 5 and 7 days post-infection suggesting that the ZIKV replication site shifted from peripheral tissues into the brain in *Stat1*^-/-^ mice. Consistent with the RNA data, ZIKV NS1 and capsid protein expression were detectable by immunoblotting in spleens harvested from mice 3 days post-infection that decreased by day 5 (Fig 1E). ZIKV NS1 protein expression in brain tissue was detected on day 7 post-infection (Fig 1F). We failed to detect viral protein expression in Day3 and Day5 brain tissue (S1 Fig). Viral protein expression in the liver was not detected by immunoblotting (S1 Fig). Similar to other flaviviruses, ZIKV NS1 protein is a secretory protein that could be used as diagnostic tool for ZIKV infection [22]. Serum NS1 expression in ZIKV-infected *Stat1*^-/-^ mice gradually increased, suggesting that systemic infection in various tissues, including the brain, continued (Fig 1G). Taken together, these data suggest that as the ZIKV infection progressed different organs were affected.

### ZKIV replication induces cell death in tissues of infected *Stat1*^-/-^ mice

**Fig 2:**
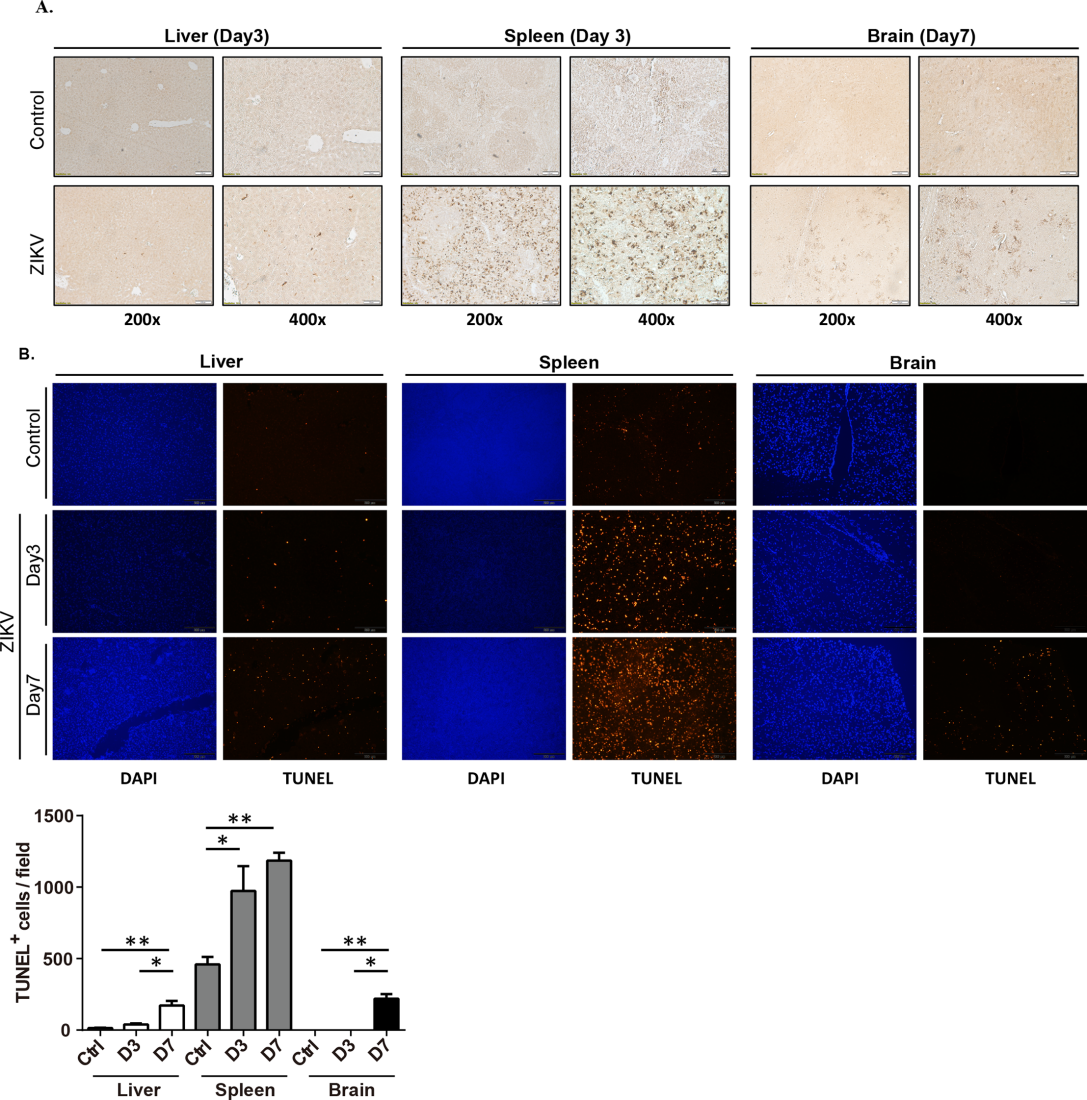
ZIKV replication and assessment of apoptosis in various organs in infected *Stat1*^-/-^ mice. **(A)** Liver, spleen, and brain tissues harvested from ZIKV-infected and uninfected *Stat1*^-/-^ mice were subjected to immunohistochemistry with an anti-NS1 antibody. **(B)** Cell death in infected tissues was determined using the TUNEL method. Tissues were collected 3 and 7 days post-infection with 4×10^4^ pfu/mouse. TUNEL^+^ signals were examined by fluorescence microscopy and images from 5–10 randomly selected 200X magnification fields were analyzed by ImageJ software (NIH).

To examine the ZIKV tissue distribution, liver, spleen, and brain sections were examined by immunohistochemistry using an anti-NS1 antibody (Fig 2A). Consistent with the mRNA expression profile (Fig 1D), NS1-expressing cells were rarely found in liver compared to the high expression levels observed in the spleen, mainly in the red pulp 3 days post-infection (Fig 2A). NS1-expressing cells were found sporadically in brain tissues examined 7 days post-infection. To examine whether ZIKV infection induced cell death, apoptosis was examined in respective tissues using the TUNEL assay. This analysis demonstrated low levels of apoptosis in the liver compared to the substantially elevated levels observed in the spleen at both 3 and 7 days post-infection (Fig 2B). The frequency of TUNEL-positive cells in the brain was mainly observed on day 7 post-infection (Fig 2B). The apoptotic cells in brain might accumulate over time and contribute to the mouse death. Taken together, ZIKV replication and cell death were observed in the spleen during the early stages of infection and in the brain at later time points.

### Proinflammatory cytokines did not play a role in ZIKV-dependent mortality

**Fig 3:**
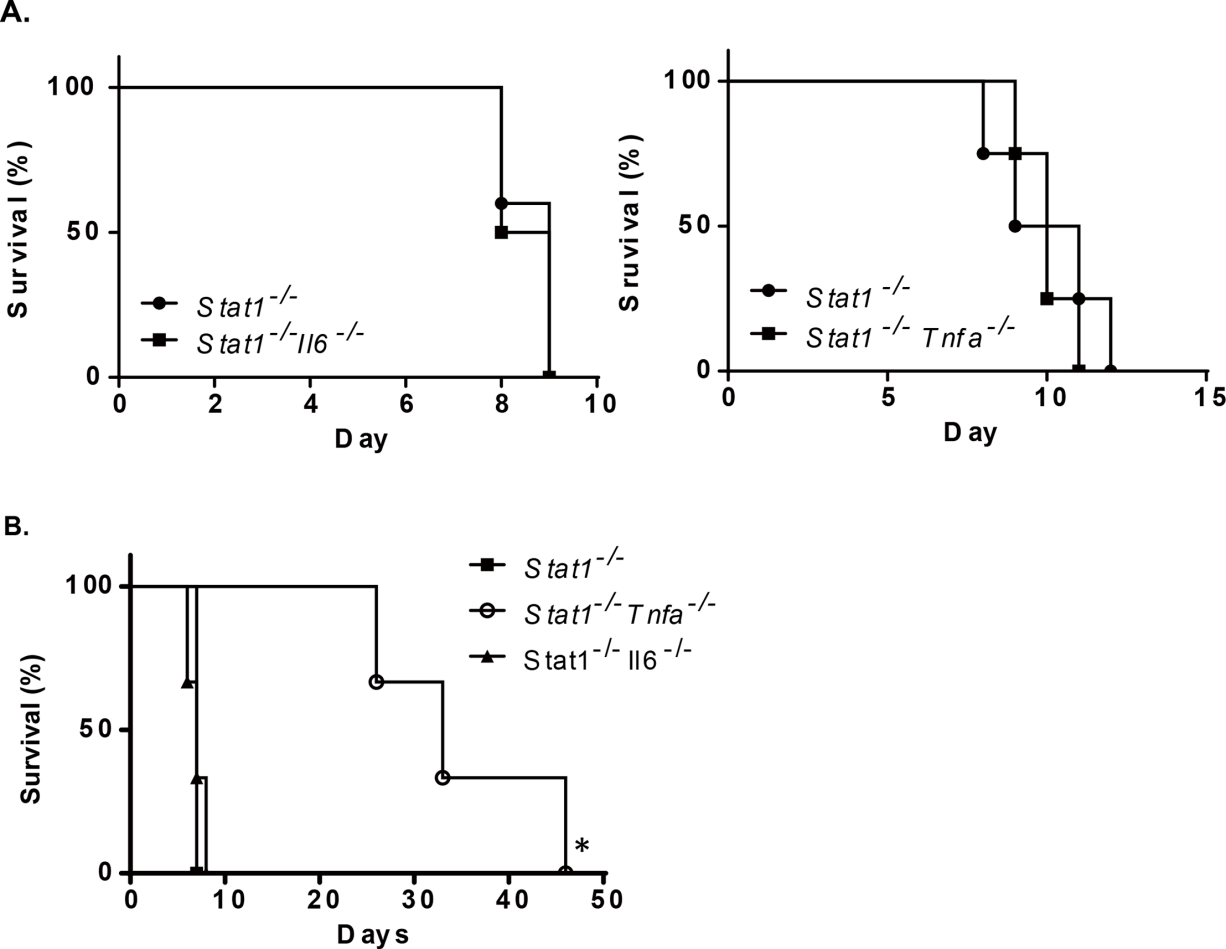
Proinflammatory cytokines were not involved in ZIKV-dependent mortality. **(A)** *Stat1*^*-/-*^, *Stat1*^*-/-*^× *Il6*^*-/-*^ and *Stat1*^*-/-*^× *Tnfa*^*-/-*^ (n = 3) were challenged with ZIKV (1×10^3^ pfu/mouse, ip) and monitored daily. **(B)** *Stat1*^-/-^, *Stat1*^-/-^× *Il6*^-/-^, and *Stat1*^-/-^× *Tnfa*^-/-^ mice were challenged with Dengue virus by intravenous injection (D2Y98P strain, 1×10^3^ pfu/mouse) and mouse survival rates were determined daily.

TNFα and IL-6 are important proinflammatory cytokines that are upregulated during acute inflammation. Dengue virus (DENV) infection induces TNFα and IL-6 production in dengue patients, and serum TNFα is positively correlated with disease progression [23]. We suspected that proinflammatory cytokines might be also involved in ZIKV-associated pathology and mouse death. To investigate the role of proinflammatory cytokines in ZIKV infection, double knockout mice (*Stat1*^*-/-*^× *Il6*^*-/-*^ and *Stat1*^*-/-*^× *Tnfa*^*-/-*^) were infected with ZIKV by direct injection. The double knockout mice were equally susceptible as *Stat1*^-/-^mice to a ZIKV infection suggesting that the upregulation of these proinflammatory cytokines might not be critical in ZIKV-induced pathology and mortality (Fig 3A). In contrast, the absence of TNFα expression delayed DENV-associated death in a mouse model (Fig 3B), suggesting that ZIKV-associated pathology develops via a different mechanism.

### *Stat1*^-/-^ mice were less competent to activate splenic cDCs and macrophages than *Ifnar*^-/-^ mice after ZIKV infection

**Fig 4:**
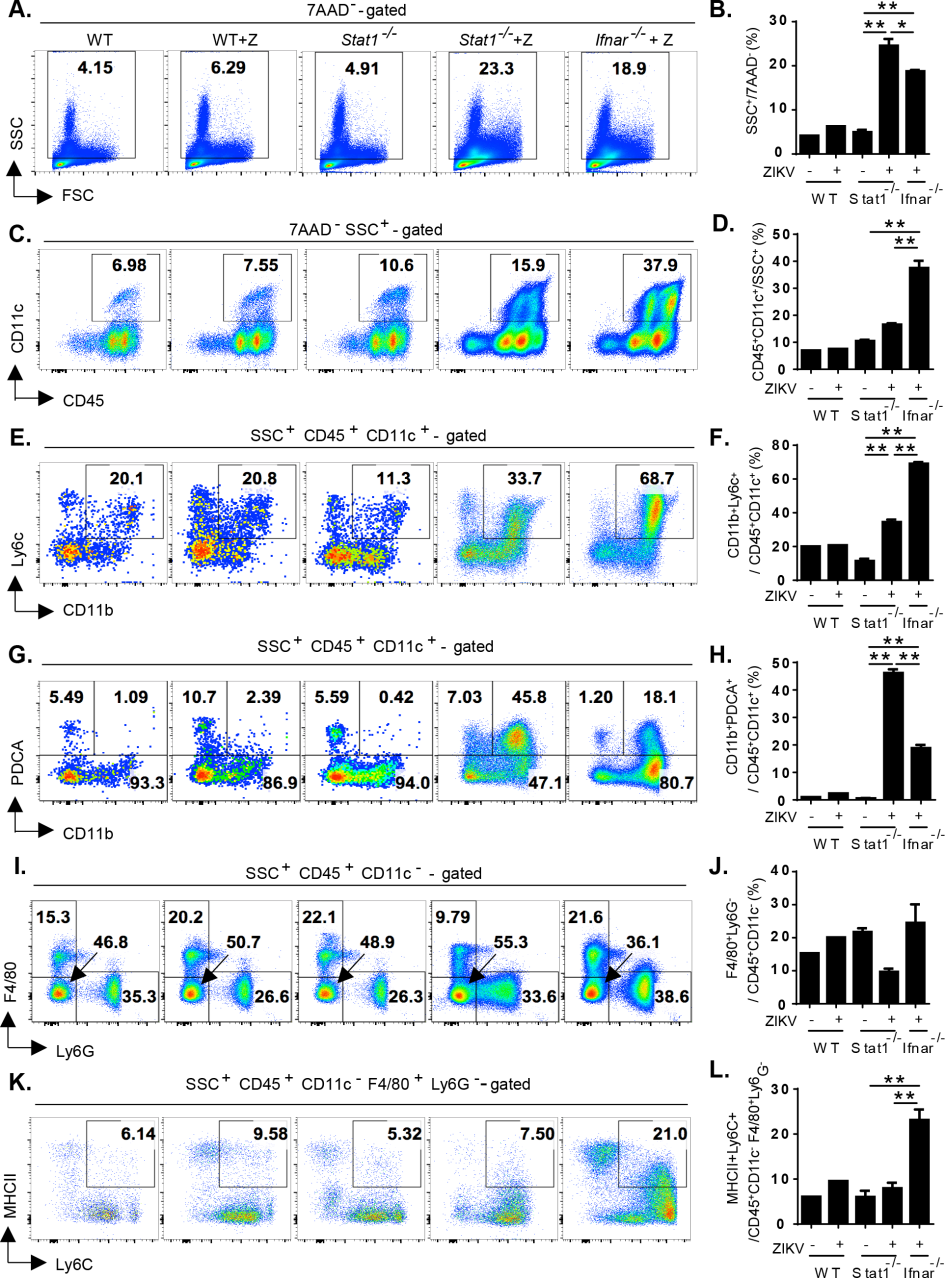
ZIKV infection caused the expansion of PDCA^+^ dendritic cells but not the activation of F4/80^+^Ly6G^-^ macrophages in *Stat1*^-/-^ mice. Splenocytes isolated from wild type (n = 1), *Stat1*^-/-^ mice (n = 3) and *Ifnar*^-/-^ mice (n = 3) without or with ZIKV infection (60 h post-infection, 4x10^4^ pfu/mouse) were subject to FACS analysis using side scattered light (SSC) for granulocyte **(A)** and specific markers for dendritic cells **(C,E,G)** and macrophage cells **(I,K)**. 7-Aminoactinomycin D (7-AAD) and CD45 were used to exclude dead and non-hematopoietic cells, respectively. For quantitation analysis, the percentage of specific subpopulations to the gated population was calculated for each splenocyte preparation. Quantifications of the subpopulations of immune cells were shown in **(B,D,F,H,J,L)**. *, p ≤ 0.05; **, p ≤ 0.01 ***Stat1***^**-/-**^ **mice were less competent to activate splenic cDCs and macrophages than *Ifnar***^**-/-**^ **mice after ZIKV infection**.

In contrast to *Stat1*^-/-^ mice, wild-type (WT) mice were resistant to a ZIKV infection. Although *Ifnar*^-/-^ adult mice were sensitive to a ZIKV infection, the infected mice recovered from the infection (Fig 1A). Due to the significant levels of ZIKV replication observed in the spleen (resulting in splenomegaly) in *Stat1*^-/-^ mice. We next addressed whether anti-ZIKV-induced immune responses determined the outcome of ZIKV infection in mice. Splenocytes were isolated from WT, *Stat1*^-/-^ and *Ifnar*^-/-^ mice without or with ZIKV infection (60 h post-infection, 4x10^4^ pfu/mouse) and analyzed by flow cytometry with various cell lineage markers (Fig 4). Dendritic cells (DCs) are known to be important for the activation of adaptive immune response. Activated DCs upregulate the expression of MHCII molecule, which presents antigen peptide to prime antigen-specific T cells. In addition, activated plasmacytoid DCs produce large amounts of type I IFN immediately after virus infection, which is critical to control viral infection [24, 25]. The amounts of SSC^+^ (side scattered light) immune cells in *Stat1*^-/-^ and *Ifnar*^-/-^ mice, that contain all myeloid lineages, were comparable to those in WT mice in the steady state (S2A Fig). Upon ZIKV infection, SSC^+^ myeloid cells were increased in both *Stat1*^*-/-*^ and *Ifnar*^*-/-*^ mice compared to those in uninfected *Stat1*^*-/-*^ mice (Fig 4A and 4B, p<0.0001). Within those myeloid cells, the percentages of CD11c^+^ DC populations were similar between uninfected WT, *Stat1*^*-/-*^ and *Ifnar*^*-/-*^ mice (S2B Fig). However, the percentage of CD11c^+^ DCs was drastically increased in *Ifnar*^*-/-*^ mice at 60 h post-ZIKV infection compared to those in infected *Stat1*^*-/-*^ mice (Fig 4C and 4D, p<0.0001). We observed that Ly6C^+^CD11b^+^ conventional DCs (cDCs) as well as PDCA1^+^CD11b^+^ pDCs were significantly induced in *Stat1*^*-/-*^ and *Ifnar*^*-/-*^ mice in frequencies after infection compared to uninfected *Stat1*^*-/-*^ mice (Fig 4E–4H), while the DCs were similar between all uninfected mice (S2C and S2D Fig). Interestingly, the induction of cDCs in infected *Ifnar*^*-/-*^ was more prominent than in *Stat1*^*-/-*^ mice, while the induction of PDCA1^+^CD11b^+^ DCs was more dominant in infected *Stat1*^*-/-*^ mice.

ZIKV has broad tissue tropism and macrophages could be one of the main targets during ZIKV infection [11, 26, 27]. We addressed F4/80^+^Ly6G^-^ and MHCII^+^ macrophages among CD11c^-^ myeloid cells and found no significant difference of macrophages in frequencies in uninfected WT, *Stat1*^*-/-*^ and *Ifnar*^*-/-*^ mice (S2E and S2F Fig). After infection, the proportion of F4/80^+^Ly6G^-^ macrophages was unchanged in WT and *Ifnar*^*-/-*^ mice but slightly reduced in *Stat1*^*-/-*^ mice (Fig 4I and 4J). Further, the amount of MHCII^+^ activated macrophages in infected *Ifnar*^*-/-*^ mice was more than those in infected *Stat1*^*-/-*^ mice (Fig 4K and 4L, p = 0.0046). In contrast, infected *Stat1*^*-/-*^ mice were unable to upregulate MHCII expression in macrophages. The results implied that infected *Stat1*^*-/-*^ mice mounted an immune response, which was less competent to induce macrophage activation. Taken together, our model system showed that cDCs and splenic macrophage were not only the important target cells by ZIKV infection, but also involved in the induction of a protective immune response against ZIKV-mediated lethality.

### Establishing a mosquito-dependent ZIKV transmission cycle in *Stat1*^-/-^ mice

**Fig 5:**
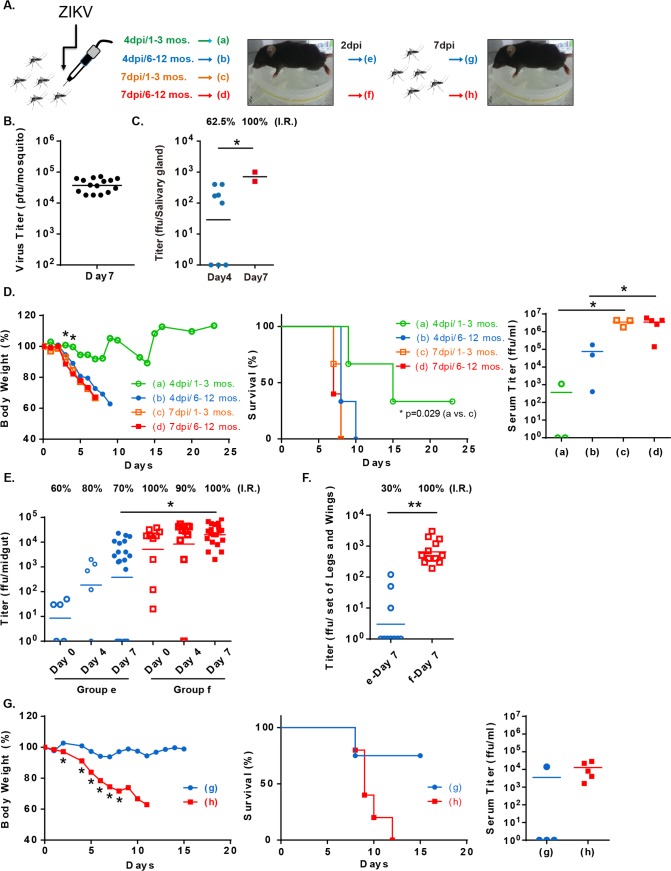
Establishing a complete mosquito-mediated ZIKV transmission cycle. **(A)** Experimental design for establishing a complete ZIKV transmission cycle. **(B)** *A*. *aegypti* mosquitoes were injected in the thorax with ZIKV (400 pfu/mosquito) and viral titers were determined by plaque assay by homogenization whole mosquitoes 7 days later (n = 15). **(C)** To confirm the infectivity, viral titers were determined by focus-forming assay by homoginizing mosquito salivary gland 4 or 7 days later (n = 8 or 2). The ZIKV-infection rate (I.R.) was calculated. **(D)** *Stat1*^-/-^ mice (n = 3) were bitten by 1–3 or 6–12 ZIKV-carrying mosquitoes (day 4 or 7 post-thoracic injection). Weights and survival were monitored daily. Virus titers in serum collected from mosquito-bitten mice day 2 post-mosquito exposure were determined by focus-forming assay. **(E)** Mosquitoes were starved overnight and allowed to take blood meals from the ZIKV-infected mice (**Group b** and **d** mice in **D**; day 2 post-ZIKV infection). The **Group e** and **f** mosquitoes took blood from **Group b** and **d** mice, respectively. Virus titers in mosquito midguts collected right after the blood meal (Day 0), 4 and 7 days later were measured by focus forming assay (n = 5–20). Infection rate was calculated. **(F)** Legs and wings collected from the **Group e** and **f** mosquitoes were also subject to virus detection (n = 10–12). **(G)** *Stat1*^-/-^ mice (**Group g** or **h**; n = 4) were bitten by the ZIKV-infected mosquitoes from **Group e** or **f**, respectively (day 7 post-infection in **E,** 6–9 mosquitoes/mouse). Weights and survival rates were monitored daily.

Based on our observations that *Stat1*^-/-^ mice infected with ZIKV were highly viremic and pathogenic, we speculated that a complete transmission cycle might be established with *Stat1*^-/-^ mice (Fig 5A). Following mosquitoes take a bloodmeal from a viremic vertebrate host, arboviruses enter and replicate in mosquito midgut lumen initially. The viruses then penetrate midgut barrier and replicate in secondary organs including salivary gland. The virus can be further transmitted through mosquito saliva to another vertebrate host [28]. To establish a mosquito-dependent transmission model, ZIKV infection were first introduced to *A*. *aegypti* mosquitoes. To have ZIKV-carrying mosquitoes, ZIKV was directly injected into the mosquito thorax (400 pfu/mosquito, Fig 5B and 5C), in which ZIKV bypass the midgut barrier [29, 30]. ZIKV replication assessed in the mosquito by plaque assay using total mosquito homogenates demonstrating that by day 7 post-infection 10^4^ to 10^5^ pfu/mosquito could be observed (Fig 5B). Viral titers in mosquito salivary gland from day 4 or 7 post-infection were also determined. As shown in Fig 5C, ZIKV was detected in the salivary gland and the infection rates on day 4 and 7 post-thoracic injection were 62.5% and 100%, respectively. Similar infection rate was also observed in the midgut (S3 Fig). The ZIKV-carrying mosquitoes were then allowed to take blood meals from *Stat1*^-/-^ mice to assess their ZIKV transmission ability. As shown in Fig 5D, *Stat1*^-/-^ mice bitten by 6–12 of Day 7 mosquitoes showed significant body weight loss and died within 10 days (Group d in Fig 5D). Bites from 1–3 of day 7 mosquitoes were also sufficient to cause mouse death (Group c in Fig 5D). When *Stat1*^-/-^ mice were exposed to the Day 4 mosquitoes, 6–12 mosquito bites also caused a mouse death in 10 days (Group b in Fig 5D). 1–3 mosquito bites caused less body weight loss and lower death rate in mice (Group a mice in Fig 5D). ZIKV was recovered from blood as early as day 2 post-mosquito exposure in those ZIKV-infected mice (Fig 5D), suggesting that the mosquitoes-to-mice transmission was successfully established.

We next determined whether ZIKV can be also transmitted from viremic *Stat1*^-/-^ mice mice to mosquitoes by allowing uninfected mosquitoes to take a blood meal from the mosquito-infected *Stat1*^-/-^ mice (Group b and d mice in Fig 5D). Engorged mosquitoes were collected and their virus titers determined 4 or 7 days later. A small portion of the engorged mosquitoes were collected immediately to evaluate the amount of virus taken from mouse blood. The mosquitoes (Group e mosquitoes in Fig 5E) which took a blood meal from mice with low ZIKV titer in blood (Group b mice in Fig 5D) indeed had lower virus replication in their midguts and reduced infection rate compared to the mosquitoes (Group f mosquitoes in Fig 5E) which took a blood meal from the mice with high virus titer in blood (Group d mice in Fig 5D). The virus dissemination of ZIKV in other body parts, such as legs and wings, on Day 7 post-infection was also evaluated. As shown in Fig 5F, the dissemination rates in Group e and f were 30% and 100%, respectively. The similar dissemination rate of ZIKV in salivary gland was observed in these mosquito groups (S4 Fig). The result suggested that ZIKV was transmitted from *Stat1*^-/-^ mice to mosquitoes and had a successful replication in the mosquitoes.

To confirm the mosquitoes that acquired their ZIKV from infected mice had virus transmission capacity to another animal, the Day 7 mosquitoes from Groups e and f mosquitoes in Fig 5E were allowed to take blood from uninfected *Stat1*^-/-^ mice (6–9 mosquitoes/mouse) (Fig 5G). All mice bitten by mosquitoes infected with high ZIKV titer blood (Group f mosquitoes in Fig 5E) had a significant weight loss and 100% died within 2 weeks (Group h mice in Fig 5G). In contrast, the mice bitten by the Day 7 mosquitoes from Group e (Fig 5E) had less weight loss and 25% death rate. The ZIKV titer in mouse blood at Day 2 post-infection was consistent with survival result (Fig 5G). Taken together, a complete ZIKV mosquitoes-to-mice-to-mosquitoes-to-mice transmission cycle was established using *Stat1*^-/-^ mice.

### Threshold of ZIKV infection in mosquito

**Fig 6:**
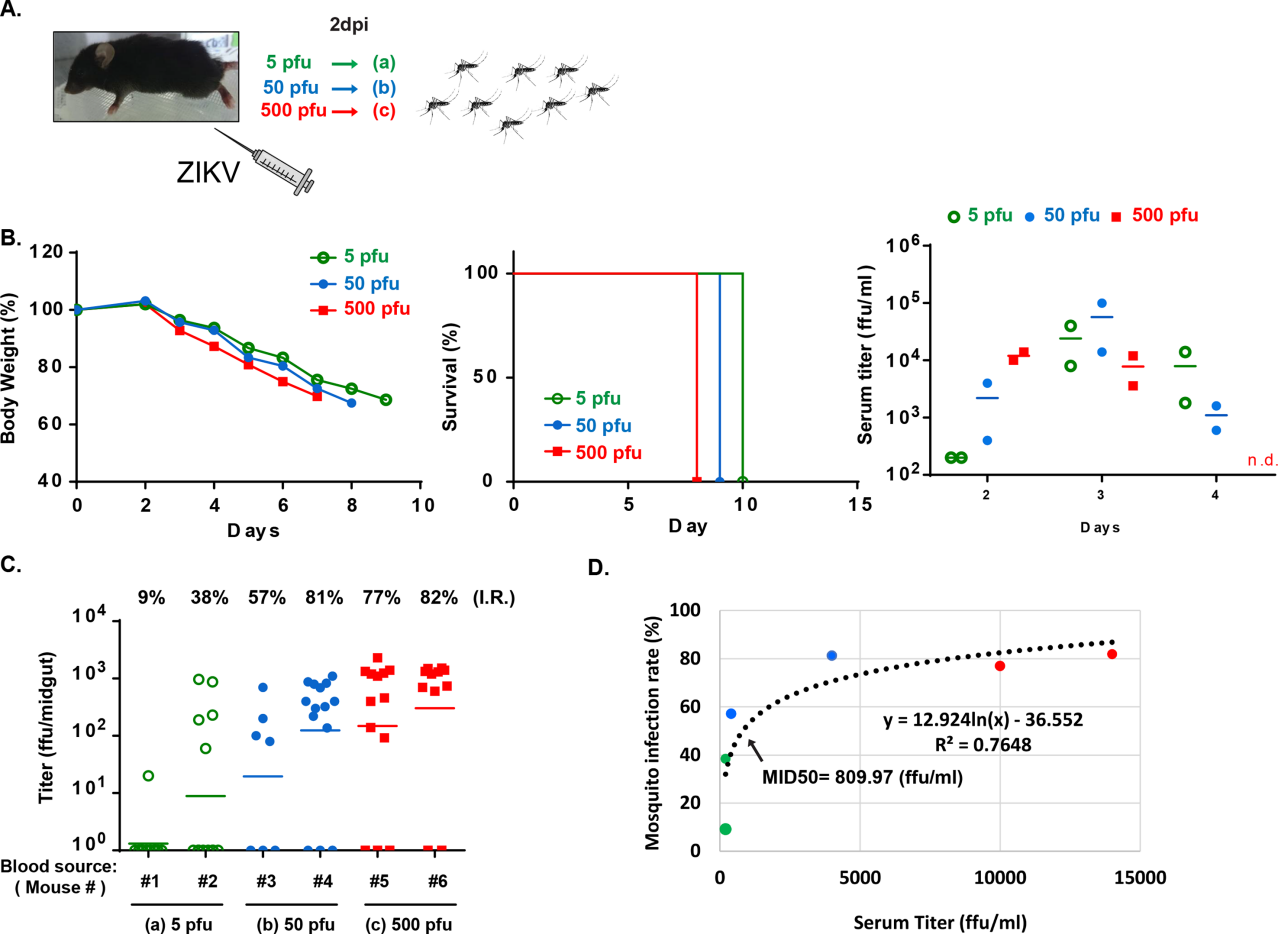
Threshold of ZIKV infection in mosquitoes. **(A)** Experimental design. **(B)** Six *Stat1*^-/-^ mice were infected with 5, 50 or 500 pfu of ZIKV intraperitoneally. Mouse body weights and survival rates were monitored daily. Virus titers in serum from day 2, 3 and 4 post-infection were determined (n = 2). **(C)** Starved mosquitoes were allowed to take blood meals from the ZIKV-infected mice (#1 to #6) in **B** on day 2 post-infection. Mosquitoes obtained blood meal from the same mouse were housed and grouped together. Virus titer and infection rate in each mosquito group were measured on day 7 post-blood meal (n = 7–14). **(D)** Based on the mouse serum data and their corresponding infection rate in mosquitoes, the mosquito infectious dose of 50% (MID50) was estimated.

We next determined the threshold of ZIKV infection in the mosquito by allowing uninfected mosquitoes to take blood meals from *Stat1*^-/-^ mice exposed to various infection doses (Fig 6A). ZIKV induced body weight loss and mouse death as low as 5 pfu of ZIKV challenge with slight changes in the kinetics of mouse viremia (Fig 6B). Mice infected with 5–500 pfu of ZIKV infection intraperitoneally had viremia with titers ranging from 200 to 14000 ffu/ml on day 2 post-infection. The mosquitoes that took blood meals from these mice had infection rates ranging from 9% to 82% (Fig 6C). Based on the data, the infectious dose at which 50% of mosquitoes become infected (MID50) by feeding on mice was estimated to be 810 ffu/ml (Fig 6D). In summary, ZIKV was highly infectious in both *Stat1*^-/-^ mice and *A*. *aegypti*.

## Discussion

In the present study, *Stat1*^*-/-*^ mice were used to establish a ZIKV infection model. *Stat1*^*-/-*^ mice were more sensitive than *Ifnar*^*-/-*^ mice to ZIKV infection without any discernable gender or age differences. With the different outcome of ZIKV infection in *Ifnar*^*-/-*^ and *Stat1*^*-/-*^ mice, cDC and macrophage activation might be the key components of protective immune responses. Furthermore, a complete ZIKV transmission cycle between mosquito and animal was established in *Stat1*^*-/-*^ mice that will be useful in the evaluation of candidate compounds targeting both vector and host.

In *Stat1*^*-/-*^ mice, ZIKV replication took place in the spleen during the early stages of infection resulting in subsequent viremia that was detectable in the blood. Consistent with the virus replication kinetics in the spleen, viral blood titers peaked on day 2 and decreased by day 3 post-infection. Serum levels of the NS1 protein remained high at later time points in *Stat1*^*-/-*^ mice, suggesting that virus replication was still ongoing in other organs. Differential kinetics of virus titer and NS1 level in serum were also observed in a recent study [31]. In contrast to the ZIKV NS1 expression pattern, DENV NS1 expression peaks on day 4 post-infection and disappears at later time points [32]. Measuring the serum levels of NS1 might be a useful biomarker in determining whether a persistent ZIKV infection is present in an epidemic area.

Although the genome structures and transmission cycles of ZIKV and DENV are very similar, the clinical presentation following infections with these viruses is different. For example, blocking TNFα ameliorates the severity of hemorrhaging caused by DENV infections [33]. In contrast, the absence of TNFα did not show any beneficial effects in ZIKV infected *Stat1*^*-/-*^ mice, suggesting that ZIKV causes pathology via different mechanisms. Whether inactivation of macrophages, insufficient activation of cDCs and excess production of PDCA1^+^CD11b^+^ DCs are directly involved in ZIKV pathogenesis warrants further investigation.

During natural infection, arboviruses enter mosquito through a bloodmeal from a viremic vertebrate host and replicates in the midgut lumen. Vector competence is determined by whether the arbovirus can penetrate the midgut and proliferate in secondary organs including salivary gland for further transmission. When entering mosquito via intrathoracic injection, viruses bypass the midgut barrier as well as the innate immunity in mosquito lumen, and therefore higher infection rate could be reached. By taking the advantage of high infection rate in mosquitoes by thoracic injection, we therefore were able to estimate other determinants, such as the numbers of mosquito bites and extrinsic incubation time, for a successful ZIKV transmission from mosquitoes to mice. To mimic nature infection in which arbovirus infection in mosquito is mediated by oral feeding from a vertebrate host, the ZIKV-infected mice were used as blood donor for mosquito infection in the second part of our transmission model. The infection rate in mosquitoes by oral feeding was dependent on the virus titer in mouse serum and was lower than the infection rate caused by intrathoracic injection. The difference between intrathoracic injection and oral feeding on virus transmission efficiency will provide a way to evaluate the influence of various factors on midgut tissue barrier and innate immune response in mosquito to a successful arbovirus transmission.

*Stat1*^*-/-*^ mice was recently applied in ZIKV research to evaluate the efficacy of ribavirin for ZIKV treatment [34]. ZIKV was highly infectious to *Stat1*^*-/-*^ mice and the lethal dose of ZIKV to the mice was even lower than 5 pfu, making the mouse strain a useful transmission model. We therefore could study not only the mosquitoes-to-mice transmission, but also the mice-to-mosquitoes transmission. ZIKV-infection caused high titer viremia which lasts at least three days in *Stat1*^*-*/-^ mice, and the MID50 to *A*. *aegypti* with the viremic mouse blood was less than 1000 ffu/ml, making the transmission mouse model very useful for future applications. For example, the transmission model could be important for establishing the extrinsic incubation period (EIP) in mosquito. From our mouse model, the EIP was ≤ 7 days and could be shorter than the A129 mice [2, 16].

The development of a novel mouse model that parallels the transmission cycle in humans will form the cornerstone of future research that will allow for a better study of disease progression and pathogenesis in addition to providing a novel means of testing developmental drugs and interventions. Additional transmission models using other mouse strains deficient in type I and II IFN signaling might also be developed as a means of further assessing immune responses to ZIKV in the context of different genetic backgrounds. Furthermore, the complete transmission model will be valuable to investigate aspects of vector competence for ZIKV and host factors that influence the extrinsic incubation period.

## Materials and methods

### Ethics statement

All mouse-related experiments were conducted in compliance with the guidelines of the Laboratory Animal Center of NHRI. The animal protocol (NHRI-IACUC-105111) was approved by the Institutional Animal Care and Use Committee of NHRI, according to the Guide for the Care and Use of Laboratory Animals (NRC 2011). Management of animal experiment and animal care and use of NHRI have accredited by the AAALAC International.

### Virus and cell culture

Zika virus strain PRVABC59 was obtained from the Taiwan Center for Disease Control and amplified in Vero cells (obtained from Dr. Min-Shi Lee, NHRI, Taiwan). Virus titers were determined by plaque assay or focus forming assay using Vero cells as described previously [35]. Dengue virus strain D2Y98P [36] was obtained from Dr. Sylvie Alonso (National University of Singapore, Singapore), amplified in C6/36 cells (obtained from Dr. Wu-Chun Tu, National Chun Hsing University, Taiwan), and titrated by plaque assay using the BHK-21 cells (obtained from Dr. Andrew Yueh, NHRI). Detailed information can be found in the Supplementary Methods.

### Antibodies, chemicals, and reagents

Antibodies to the ZIKV capsid (GeneTex, Hsinchu, Taiwan) and tubulin (Sigma, St. Louis, MO) were used for immunoblotting. ZIKV NS1 (172–351) antiserum was raised in mice and rabbits at NHRI as described in the Supplementary methods and used for immunoblotting and immunohistochemistry, and ELISA assays. Apoptosis in tissue sections was detected using the In Situ Cell Death detection kit (Roche, Indianapolis, IN).

### Mosquitoes

*Aedes aegypti* (Higgs) eggs were hatched in deionized water under deoxygenated conditions. The hatched larvae were fed powdered yeast and goose liver (NTN Fishing Bait). Newly emerged mosquitoes were fed with a 10% sucrose solution and maintained at 28°C and 80% humidity with a 12 h light and dark cycle. For direct ZIKV infection, 7–14 day-old female mosquitoes were inoculated with 400 pfu of ZKIV by thoracic injection and maintained in normal housing conditions for 7 days.

### Mice

Mice were bred and maintained at the Laboratory Animal Center of NHRI. *Stat1*^-/-^ (C57BL/6 background) [18] mice were provided by Dr. Chien-Kuo Lee (NTU, Taiwan). Wild-type C57BL/6, *Il6*^*-/-*^, and *Tnfa*^*-/-*^ mice were purchased from the Jackson Laboratory. *Stat1*^-/-^ mice were crossed with *Il6*^*-/-*^and *Tnfa*^*-/-*^ mice to establish double-knockout mice. *Ifnar*^*-/-*^ mice (C57BL/6 background) were provided by Dr. Michael Karin (UCSD, US). Unless otherwise specified, both male and female mice between the ages of 8–10 weeks were used in the study.

### Mosquito-mediated ZIKV mouse infection

Mice were anesthetized with Rompun (16 mg/Kg, Bayer Animal Health, Monheim, Germany) and Ketalar (100 mg/Kg, Pfizer, New York, NY). After their ventral surfaces were shaved they were placed on top of a polyester mesh that covered a mosquito-housing cage that allowed female mosquitoes to take a blood meal. Female mosquitoes were starved for 12 h before they were allowed to take blood meals from mice. Each mouse was bitten by 6–12 mosquitoes. The ZIKV-infected mosquitoes were kept in a separated mosquito incubator and maintained at 28°C and 80% humidity with a 12 h light and dark cycle.

### ZIKV NS1 ELISA

NS1 (172–351) rabbit antiserum was used as a capture antibody (10 μg IgG/well) and mouse antiserum was used as the detection antibody (2 μg IgG/well). Anti-mouse-HRP (1:5000, Santa Cruz, Santa Cruz, CA) and TMB (eBioscience) were used for color development. Purified NS1 (172–352) protein was used as a standard (1–1000 ng/ml) to calculate NS1 serum and culture media concentrations.

### Quantitative real-time (RT)-PCR

RNA was extracted from mouse tissues using Trizol (Invitrogen, Carlsbad, CA) and subsequently used as templates for cDNA synthesis using SuperScript First Strand Synthesis System (Invitrogen). cDNAs were then used as templates in the quantitative-polymerase chain reaction (PCR) with gene specific primers and SYBR green dye to determine quantification cycle (Cq) by Applied Biosystems 7900HT Fast Real-Time PCR System. Relative ZIKV expression level was calculated using the ΔΔCq method with cyclophilin A (CPH) cDNA as an internal control. Primer sequences used in the study were: CPH Forward: atggtcaaccccaccgtgt, Reverse: ttcttgctgtctttggaactttgtc; ZIKV(1086–1162) [37] Forward: ccgctgcccaacacaag, Reverse: ccactaacgttcttttgcagacat.

### Flow cytometry

Spleens were mashed and digested with Liberase Blenzyme 3 (0.05U/ml, Roche, Woerden, Netherlands) plus DNaseI (10 μg/ml, Sigma) at 37°C for 20 min. Single cell suspensions of 1x10^6^ cells were washed twice with FACS buffer (2% BSA/PBS, 0.1% NaN_3_) and maintained in the dark at 4°C throughout experiments. Flow cytometric data were acquired using a CantoII flow cytometer and FACSDiva software (both from BD Biosciences, San Jose, CA). FlowJo software (Tree Star, Inc.) was used for data analyses. For marker expression determinations, cells were incubated for 15 min on ice with anti-mouse antibodies, including CD11b (M1/70), CD11c (HL3), CD45 (30-F11), F4/80 (BM8), Ly6G (IA8), Ly6C (AL-21) from BD Biosciences and PDCA1 (eBio129c) from eBioscience. Immunostained cells were washed twice in FACS buffer prior to incubation with 7AAD (Sigma). Viable cells were gated from the 7AAD-negative population prior to analysis. For quantitation analysis, the percentage of specific subpopulations to the gated population was calculated in each splenocyte preparation.

### Statistical analysis

Unless otherwise stated, data are presented as the mean ± SEM. The difference among treatment or day groups were analyzed using one-way ANOVA with Bonferroni’s post hoc test, or Student's T test. The results of survival curve were analyzed by log-rank test. p-values ≤0.05 were considered statistically significant. The exact p, t, and df values can be found in S1 Table.

## Supporting information

S1 Methods Cell culture.Plaque forming assay.Focus forming assay.ZIKV NS1 (172–351) rabbit antiserum preparation.Immunohistochemistry.TUNEL assay.
(DOCX)

S1 Fig ZIKV protein expression was absent in the early phase in brain and was not detected in liver throughout the infection time. ZIKV NS1 protein expression in brains and livers harvested at D3 and D5 post-infection (4×10^4^ pfu/mouse) and D7 post-infection (1×10^3^ pfu/mouse) were examined by immunoblotting with specific antibodies for NS1.
(TIF)

S2 Fig Subpopulations of splenocytes were similar in WT, *Stat1*^-/-^ and *Ifnar*^-/-^ naïve mice. Splenocytes isolated from naïve mice were subjected to FACS analysis using side scattered light (SSC) for granulocyte **(A)** and specific markers for dendritic cells **(B-D)** and macrophage cells **(E,F)**. 7-Aminoactinomycin D (7-AAD) and CD45 were used to exclude dead and non-hematopoietic cells, respectively.
(TIF)

S3 Fig Virus titer and infection rate in salivary gland post-thoracic ZIKV injection. *A*. *aegypti* mosquitoes were injected in the thorax with ZIKV (400 pfu/mosquito) and viral titers were determined by focus-forming assay by homoginizing mosquito midgut 4 or 7 days later (n = 20).The ZIKV-infection rate (I.R.) was calculated.
(TIF)

S4 Fig Virus titer and infection rate in salivary gland post-blood meal. The salivary glands of the Group e and f mosquitoes which took blood meals from the ZIKV-infected mice (Group b and d mice, respectively, in Fig 5D; day 2 post-ZIKV infection) were isolated right after blood meal or on Day 4 or 7 post-blood meal and subject to virus titration by focus forming assay (n = 3–4). Infection rate was calculated.
(TIF)

S1 Table The p,t and df values of the significant difference in Figures. (DOCX)

## References

[1] Guerbois M, Fernandez-Salas I, Azar SR, Danis-Lozano R, Alpuche-Aranda CM, Leal G, et al Outbreak of Zika Virus Infection, Chiapas State, Mexico, 2015, and First Confirmed Transmission by Aedes aegypti Mosquitoes in the Americas. J Infect Dis. 2016;214(9):1349–56. doi: 10.1093/infdis/jiw302 ; PubMed Central PMCID: PMCPMC5079363. 27436433 10.1093/infdis/jiw302 PMC5079363

[2] Roundy CM, Azar SR, Rossi SL, Huang JH, Leal G, Yun R, et al Variation in Aedes aegypti Mosquito Competence for Zika Virus Transmission. Emerg Infect Dis. 2017;23(4):625–32. doi: 10.3201/eid2304.161484 ; PubMed Central PMCID: PMCPMC5367433. 28287375 10.3201/eid2304.161484 PMC5367433

[3] Campos GS, Bandeira AC, Sardi SI. Zika Virus Outbreak, Bahia, Brazil. Emerg Infect Dis. 2015;21(10):1885–6. doi: 10.3201/eid2110.150847 ; PubMed Central PMCID: PMCPMC4593454. 26401719 10.3201/eid2110.150847 PMC4593454

[4] Mlakar J, Korva M, Tul N, Popovic M, Poljsak-Prijatelj M, Mraz J, et al Zika Virus Associated with Microcephaly. The New England journal of medicine. 2016;374(10):951–8. doi: 10.1056/NEJMoa1600651 . 26862926 10.1056/NEJMoa1600651

[5] Schuler-Faccini L, Ribeiro EM, Feitosa IM, Horovitz DD, Cavalcanti DP, Pessoa A, et al Possible Association Between Zika Virus Infection and Microcephaly—Brazil, 2015. MMWR Morb Mortal Wkly Rep. 2016;65(3):59–62. doi: 10.15585/mmwr.mm6503e2 . 26820244 10.15585/mmwr.mm6503e2

[6] Martines RB, Bhatnagar J, Keating MK, Silva-Flannery L, Muehlenbachs A, Gary J, et al Notes from the Field: Evidence of Zika Virus Infection in Brain and Placental Tissues from Two Congenitally Infected Newborns and Two Fetal Losses—Brazil, 2015. MMWR Morb Mortal Wkly Rep. 2016;65(6):159–60. doi: 10.15585/mmwr.mm6506e1 . 26890059 10.15585/mmwr.mm6506e1

[7] Oehler E, Watrin L, Larre P, Leparc-Goffart I, Lastere S, Valour F, et al Zika virus infection complicated by Guillain-Barre syndrome—case report, French Polynesia, December 2013. Euro Surveill. 2014;19(9). . 24626205 10.2807/1560-7917.es2014.19.9.20720

[8] Cao-Lormeau VM, Blake A, Mons S, Lastere S, Roche C, Vanhomwegen J, et al Guillain-Barre Syndrome outbreak associated with Zika virus infection in French Polynesia: a case-control study. Lancet. 2016;387(10027):1531–9. doi: 10.1016/S0140-6736(16)00562-6 . 26948433 10.1016/S0140-6736(16)00562-6 PMC5444521

[9] Hills SL, Russell K, Hennessey M, Williams C, Oster AM, Fischer M, et al Transmission of Zika Virus Through Sexual Contact with Travelers to Areas of Ongoing Transmission—Continental United States, 2016. MMWR Morb Mortal Wkly Rep. 2016;65(8):215–6. doi: 10.15585/mmwr.mm6508e2 . 26937739 10.15585/mmwr.mm6508e2

[10] D'Ortenzio E, Matheron S, Yazdanpanah Y, de Lamballerie X, Hubert B, Piorkowski G, et al Evidence of Sexual Transmission of Zika Virus. The New England journal of medicine. 2016;374(22):2195–8. doi: 10.1056/NEJMc1604449 . 27074370 10.1056/NEJMc1604449

[11] Lazear HM, Govero J, Smith AM, Platt DJ, Fernandez E, Miner JJ, et al A Mouse Model of Zika Virus Pathogenesis. Cell host & microbe. 2016;19(5):720–30. doi: 10.1016/j.chom.2016.03.010 ; PubMed Central PMCID: PMC4866885. 27066744 10.1016/j.chom.2016.03.010 PMC4866885

[12] Rossi SL, Tesh RB, Azar SR, Muruato AE, Hanley KA, Auguste AJ, et al Characterization of a Novel Murine Model to Study Zika Virus. Am J Trop Med Hyg. 2016;94(6):1362–9. doi: 10.4269/ajtmh.16-0111 ; PubMed Central PMCID: PMCPMC4889758. 27022155 10.4269/ajtmh.16-0111 PMC4889758

[13] Tripathi S, Balasubramaniam VR, Brown JA, Mena I, Grant A, Bardina SV, et al A novel Zika virus mouse model reveals strain specific differences in virus pathogenesis and host inflammatory immune responses. PLoS Pathog. 2017;13(3):e1006258 doi: 10.1371/journal.ppat.1006258 ; PubMed Central PMCID: PMCPMC5373643. 28278235 10.1371/journal.ppat.1006258 PMC5373643

[14] Morrison TE, Diamond MS. Animal Models of Zika Virus Infection, Pathogenesis, and Immunity. Journal of virology. 2017 doi: 10.1128/JVI.00009-17 . 28148798 10.1128/JVI.00009-17 PMC5375682

[15] Pingen M, Bryden SR, Pondeville E, Schnettler E, Kohl A, Merits A, et al Host Inflammatory Response to Mosquito Bites Enhances the Severity of Arbovirus Infection. Immunity. 2016;44(6):1455–69. doi: 10.1016/j.immuni.2016.06.002 ; PubMed Central PMCID: PMCPMC4920956. 27332734 10.1016/j.immuni.2016.06.002 PMC4920956

[16] Azar SR, Roundy CM, Rossi SL, Huang JH, Leal G, Yun R, et al Differential Vector Competency of Aedes albopictus Populations from the Americas for Zika Virus. Am J Trop Med Hyg. 2017;97(2):330–9. doi: 10.4269/ajtmh.16-0969 . 28829735 10.4269/ajtmh.16-0969 PMC5544086

[17] Secundino NFC, Chaves BA, Orfano AS, Silveira KRD, Rodrigues NB, Campolina TB, et al Zika virus transmission to mouse ear by mosquito bite: a laboratory model that replicates the natural transmission process. Parasit Vectors. 2017;10(1):346 doi: 10.1186/s13071-017-2286-2 ; PubMed Central PMCID: PMCPMC5520231. 28728607 10.1186/s13071-017-2286-2 PMC5520231

[18] Durbin JE, Hackenmiller R, Simon MC, Levy DE. Targeted disruption of the mouse Stat1 gene results in compromised innate immunity to viral disease. Cell. 1996;84(3):443–50. Epub 1996/02/09. S0092-8674(00)81289-1 [pii]. . 8608598 10.1016/s0092-8674(00)81289-1

[19] Grant A, Ponia SS, Tripathi S, Balasubramaniam V, Miorin L, Sourisseau M, et al Zika Virus Targets Human STAT2 to Inhibit Type I Interferon Signaling. Cell host & microbe. 2016;19(6):882–90. doi: 10.1016/j.chom.2016.05.009 ; PubMed Central PMCID: PMCPMC4900918. 27212660 10.1016/j.chom.2016.05.009 PMC4900918

[20] Bowen JR, Quicke KM, Maddur MS, O'Neal JT, McDonald CE, Fedorova NB, et al Zika Virus Antagonizes Type I Interferon Responses during Infection of Human Dendritic Cells. PLoS Pathog. 2017;13(2):e1006164 doi: 10.1371/journal.ppat.1006164 ; PubMed Central PMCID: PMCPMC5289613. 28152048 10.1371/journal.ppat.1006164 PMC5289613

[21] Kumar A, Hou S, Airo AM, Limonta D, Mancinelli V, Branton W, et al Zika virus inhibits type-I interferon production and downstream signaling. EMBO Rep. 2016;17(12):1766–75. doi: 10.15252/embr.201642627 ; PubMed Central PMCID: PMCPMC5283583. 27797853 10.15252/embr.201642627 PMC5283583

[22] Steinhagen K, Probst C, Radzimski C, Schmidt-Chanasit J, Emmerich P, van Esbroeck M, et al Serodiagnosis of Zika virus (ZIKV) infections by a novel NS1-based ELISA devoid of cross-reactivity with dengue virus antibodies: a multicohort study of assay performance, 2015 to 2016. Euro Surveill. 2016;21(50). doi: 10.2807/1560-7917.ES.2016.21.50.30426 ; PubMed Central PMCID: PMCPMC5291135. 28006649 10.2807/1560-7917.ES.2016.21.50.30426 PMC5291135

[23] Hober D, Poli L, Roblin B, Gestas P, Chungue E, Granic G, et al Serum levels of tumor necrosis factor-alpha (TNF-alpha), interleukin-6 (IL-6), and interleukin-1 beta (IL-1 beta) in dengue-infected patients. Am J Trop Med Hyg. 1993;48(3):324–31. . 8470771 10.4269/ajtmh.1993.48.324

[24] Siegal FP, Kadowaki N, Shodell M, Fitzgerald-Bocarsly PA, Shah K, Ho S, et al The nature of the principal type 1 interferon-producing cells in human blood. Science. 1999;284(5421):1835–7. . 10364556 10.1126/science.284.5421.1835

[25] Asselin-Paturel C, Boonstra A, Dalod M, Durand I, Yessaad N, Dezutter-Dambuyant C, et al Mouse type I IFN-producing cells are immature APCs with plasmacytoid morphology. Nat Immunol. 2001;2(12):1144–50. doi: 10.1038/ni736 . 11713464 10.1038/ni736

[26] Quicke KM, Bowen JR, Johnson EL, McDonald CE, Ma H, O'Neal JT, et al Zika Virus Infects Human Placental Macrophages. Cell host & microbe. 2016;20(1):83–90. doi: 10.1016/j.chom.2016.05.015 ; PubMed Central PMCID: PMCPMC5166429. 27247001 10.1016/j.chom.2016.05.015 PMC5166429

[27] Lum FM, Low DK, Fan Y, Tan JJ, Lee B, Chan JK, et al Zika Virus Infects Human Fetal Brain Microglia and Induces Inflammation. Clin Infect Dis. 2017;64(7):914–20. doi: 10.1093/cid/ciw878 . 28362944 10.1093/cid/ciw878

[28] Franz AW, Kantor AM, Passarelli AL, Clem RJ. Tissue Barriers to Arbovirus Infection in Mosquitoes. Viruses. 2015;7(7):3741–67. doi: 10.3390/v7072795 ; PubMed Central PMCID: PMCPMC4517124. 26184281 10.3390/v7072795 PMC4517124

[29] Moreira LA, Iturbe-Ormaetxe I, Jeffery JA, Lu G, Pyke AT, Hedges LM, et al A Wolbachia symbiont in Aedes aegypti limits infection with dengue, Chikungunya, and Plasmodium. Cell. 2009;139(7):1268–78. doi: 10.1016/j.cell.2009.11.042 . 20064373 10.1016/j.cell.2009.11.042

[30] Zhu Y, Zhang R, Zhang B, Zhao T, Wang P, Liang G, et al Blood meal acquisition enhances arbovirus replication in mosquitoes through activation of the GABAergic system. Nat Commun. 2017;8(1):1262 doi: 10.1038/s41467-017-01244-6 ; PubMed Central PMCID: PMCPMC5665997. 29093445 10.1038/s41467-017-01244-6 PMC5665997

[31] Liu Y, Liu J, Du S, Shan C, Nie K, Zhang R, et al Evolutionary enhancement of Zika virus infectivity in Aedes aegypti mosquitoes. Nature. 2017 doi: 10.1038/nature22365 . 28514450 10.1038/nature22365 PMC5885636

[32] Watanabe S, Tan KH, Rathore AP, Rozen-Gagnon K, Shuai W, Ruedl C, et al The magnitude of dengue virus NS1 protein secretion is strain dependent and does not correlate with severe pathologies in the mouse infection model. J Virol. 2012;86(10):5508–14. doi: 10.1128/JVI.07081-11 ; PubMed Central PMCID: PMCPMC3347314. 22419801 10.1128/JVI.07081-11 PMC3347314

[33] Chen HC, Hofman FM, Kung JT, Lin YD, Wu-Hsieh BA. Both virus and tumor necrosis factor alpha are critical for endothelium damage in a mouse model of dengue virus-induced hemorrhage. J Virol. 2007;81(11):5518–26. Epub 2007/03/16. JVI.02575-06 [pii] doi: 10.1128/JVI.02575-06 ; PubMed Central PMCID: PMC1900309. 17360740 10.1128/JVI.02575-06 PMC1900309

[34] Kamiyama N, Soma R, Hidano S, Watanabe K, Umekita H, Fukuda C, et al Ribavirin inhibits Zika virus (ZIKV) replication in vitro and suppresses viremia in ZIKV-infected STAT1-deficient mice. Antiviral Res. 2017;146:1–11. doi: 10.1016/j.antiviral.2017.08.007 . 28818572 10.1016/j.antiviral.2017.08.007 PMC7113888

[35] Hsu AY, Wu SR, Tsai JJ, Chen PL, Chen YP, Chen TY, et al Infectious dengue vesicles derived from CD61+ cells in acute patient plasma exhibited a diaphanous appearance. Sci Rep. 2015;5:17990 doi: 10.1038/srep17990 ; PubMed Central PMCID: PMCPMC4675971. 26657027 10.1038/srep17990 PMC4675971

[36] Tan GK, Ng JK, Trasti SL, Schul W, Yip G, Alonso S. A non mouse-adapted dengue virus strain as a new model of severe dengue infection in AG129 mice. PLoS Negl Trop Dis. 2010;4(4):e672 doi: 10.1371/journal.pntd.0000672 ; PubMed Central PMCID: PMC2860513. 20436920 10.1371/journal.pntd.0000672 PMC2860513

[37] Lanciotti RS, Kosoy OL, Laven JJ, Velez JO, Lambert AJ, Johnson AJ, et al Genetic and serologic properties of Zika virus associated with an epidemic, Yap State, Micronesia, 2007. Emerg Infect Dis. 2008;14(8):1232–9. doi: 10.3201/eid1408.080287 ; PubMed Central PMCID: PMCPMC2600394. 18680646 10.3201/eid1408.080287 PMC2600394

