# Supplementary material for: Establishment of a mouse model for the complete mosquito-mediated transmission cycle of Zika virus
Source: PLoS Negl Trop Dis. 2018 Apr 18;12(4):e0006417. doi: 10.1371/journal.pntd.0006417 (PMC5927462; doi:10.1371/journal.pntd.0006417)
Supplement: S1 Methods — Cell culture.Plaque forming assay.Focus forming assay.ZIKV NS1 (172–351) rabbit antiserum preparation.Immunohistochemistry.TUNEL assay. (DOCX) [file pntd.0006417.s001.docx]

**Supplementary Methods**

1. **Cell culture.** C6/36 cells were grown at 28°C in 1X Leibovitz's L-15 medium supplemented with 10% fetal bovine serum (FBS), 1X MEM Non-Essential Amino Acids Solution, and 1X Antibiotic-Antimycotic. Vero and BHK-21 cells were both grown in 1X Dulbecco's Modified Eagle Medium (DMEM) supplemented with 2% FBS and 1X Antibiotic-Antimycotic at 37 °C. All the reagents were purchased from Thermo Fisher Scientific.
2. **Plaque forming assay.** Virus titer in cell culture supernatant, mouse serum and mosquito homogenate was determined by plaque assay as described before [1]. In brief, the virus-containing samples were serially diluted in serum-free DMEM medium and added to Vero or BHK-21 monolayer cells for virus absorption at 37 °C for 2 hours. After diluted samples were removed, the cells were overlaid with DMEM containing 1% methylcellulose (4000 cps, Sigma-Aldrich), 2 % FBS, 2mM glutamine, 1mM sodium pyruvate, 2.5mM HEPES, 1X penicillin-streptomycin and cultured at 37 °C for 4 days for Vero cells and 5 days for BHK cells, respectively. The overlay medium was then gently removed, and the cells were stained with Rapid Gram Stain solution (Tonyar Biotech) for 2 hours and washed with tap water. Plaques were counted and virus titer was determined as plaque forming units per milliliter (pfu/mL).
3. **Colorimetric Focus-forming assay.** Virus titer in cell culture supernatant, mouse serum and mosquito homogenate was determined by colorimetric Focus-forming assay. In brief, virus-containing samples were serially diluted in serum-free DMEM medium and added to Vero cell for virus absorption at 37 °C for 3 hours. After diluted samples were removed, the cells were overlaid with DMEM containing 0.8% methylcellulose (4000 cps, Sigma-Aldrich), 2 % FBS, 2mM glutamine, 1mM sodium pyruvate, 2.5mM HEPES, 1X penicillin-streptomycin and cultured at 37 °C for 3 days. After being fixed with 3.7% formaldehyde for 15min, infected cells were permeabilized with 0.1%NP40 in PBS (15 min) and incubated in blocking buffer (3%BSA in PBS, 15min). Then the cells were incubated with Mouse anti-flavivirus group antigen (D1-4G2-4-15ATCC^®^ HB-112^™^) (1:5000) at 37°C 1hr, followed by incubation with goat anti-mouse secondary antibody conjugated to horseradish peroxidase (1:50000) at room temperature for 1 hr. TMB (KPL 50-77-40) were used for color development at room temperature for 20 min. Virus titer was calculated as focus-forming unit (ffu).
4. **ZIKV NS1 (172-351) rabbit antiserum preparation.** The DNA fragment of ZIKV NS1 encoding C-terminal 172-351 amino acid residues was amplified by PCR with specific primers from cDNA of ZIKV (PRVABC59) RNA genome and cloned into a pET-15b vector (Novagen) to express His-tagged NS1(172-351) recombinant protein in E coli. The recombinant His-tagged NS1(172-351) protein was purified from inclusion body with Ni-NTA column (GE Healthcare) in denaturing condition (6M guanidine hydrochloride, 50mM Tris-HCl, pH8) and refolded in refolding buffer (100 mM Tris-HCl pH 8.0, 2 mM EDTA, 400 mM L-arginine, 0.5 mM oxidized glutathione and 5 mM reduced glutathione) at 4℃overnight as described previously [2]. Solubilized NS1(172-351) protein was mixed with CpG DNA and Freund's Adjuvant to immunize mouse and Rabbit based on the immunization protocol developed by Dr. Tsung-Hsien Chuang (NHRI, Taiwan). The serum of the animals after three times of immunization was collected. The sensitivity and specificity for NS1protein detection of the antiserum was carefully conformed.
5. **Immunohistochemistry.** Livers, spleen and brain collected from mice were fixed in 10% buffered formalin, preserved in paraffin blocks and sectioned. Paraffin-embedded sections were rehydrated using a standard procedure and subjected to antigen retrieval with citrate buffer. After 3% H_2_O_2_ and 1% BSA blocking, the sections were incubated with rabbit anti-NS1 (172-351) rabbit antiserum (1:1000) at 4°C overnight, followed by incubation with a secondary antibody (EnVision^+^ system-HRP labeled polymer, DakoCytomation) at room temperature for 1 hr. The sections were then incubated with DAB substrate for color development, counterstained with hematoxylin. Serial sections were also stained with Hematoxylin and Eosin in parallel at pathology core lab at National Health Research Institutes.
6. **TUNEL assay.** Apoptotic cell death analysis: Apoptotic cell death was examined by In Situ Cell Death Detection Kit (Roche) on paraffin-embedded tissue sections by following the manufacturer’s instructions.  TUNEL^+^ signals were examined by fluorescence microscopy and images from five randomly selected fields were analyzed by ImageJ software (NIH).

**Reference**

1. Hsu AY, Wu SR, Tsai JJ, Chen PL, Chen YP, Chen TY, et al. Infectious dengue vesicles derived from CD61+ cells in acute patient plasma exhibited a diaphanous appearance. Scientific reports. 2015;5:17990. Epub 2015/12/15. doi: 10.1038/srep17990. PubMed PMID: 26657027; PubMed Central PMCID: PMC4675971.

2. Song H, Qi J, Haywood J, Shi Y, Gao GF. Zika virus NS1 structure reveals diversity of electrostatic surfaces among flaviviruses. Nat Struct Mol Biol. 2016;23(5):456-8. doi: 10.1038/nsmb.3213. PubMed PMID: 27088990.
