# Supplementary figures and images for: Establishment of a mouse model for the complete mosquito-mediated transmission cycle of Zika virus
Source: PLoS Negl Trop Dis. 2018 Apr 18;12(4):e0006417. doi: 10.1371/journal.pntd.0006417 (PMC5927462; doi:10.1371/journal.pntd.0006417)

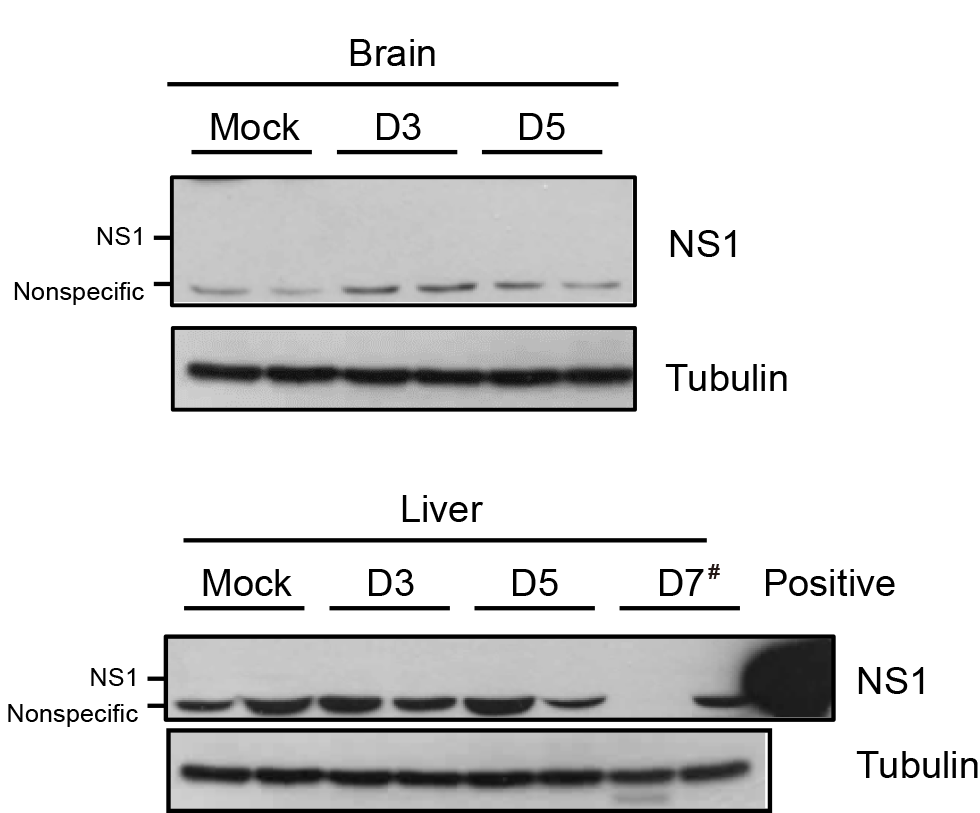

Supplement: S1 Fig — ZIKV NS1 protein expression in brains and livers harvested at D3 and D5 post-infection (4×10^4^ pfu/mouse) and D7 post-infection (1×10^3^ pfu/mouse) were examined by immunoblotting with specific antibodies for NS1. (TIF) [file pntd.0006417.s002.tif]

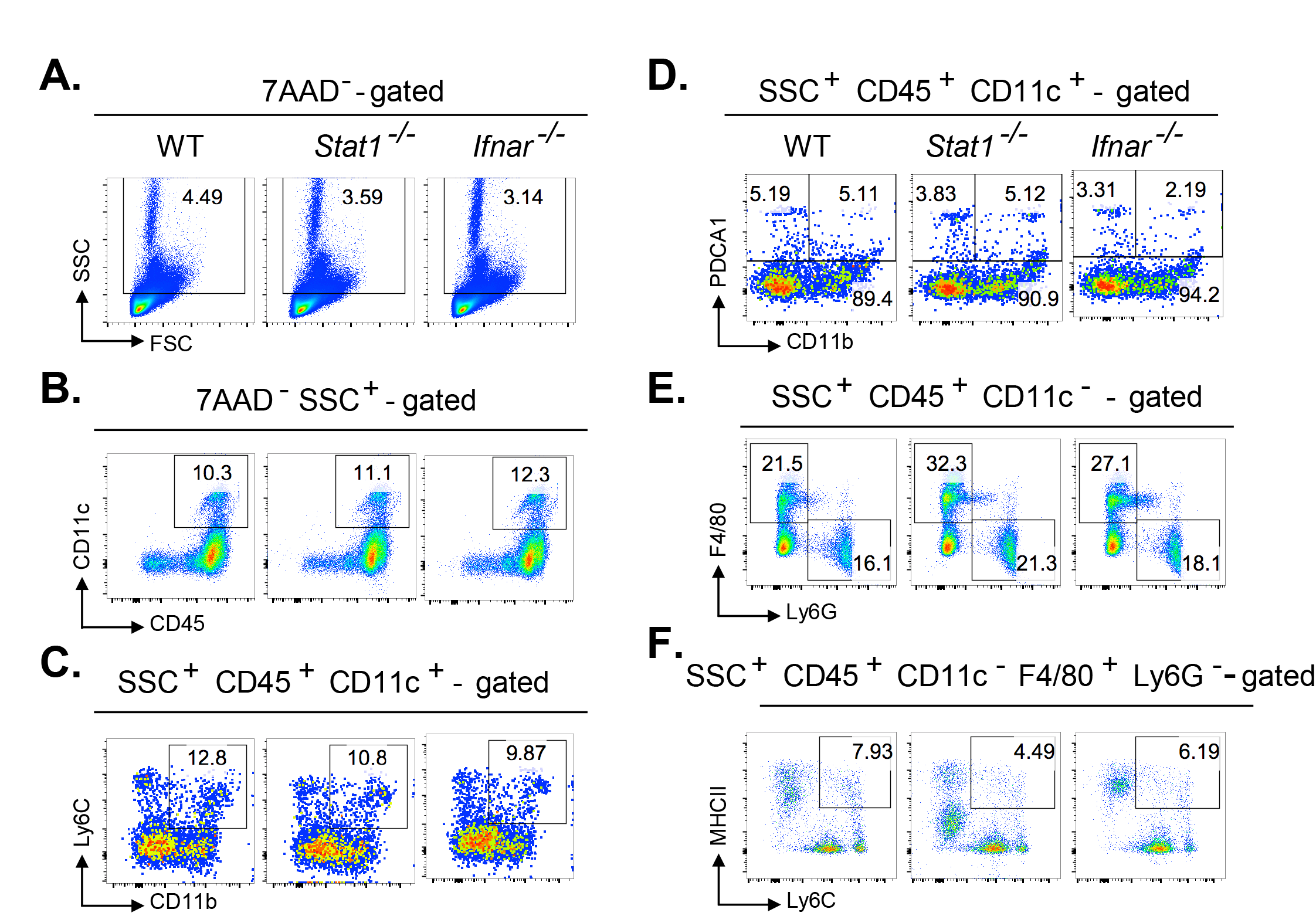

Supplement: S2 Fig — Splenocytes isolated from naïve mice were subjected to FACS analysis using side scattered light (SSC) for granulocyte (A) and specific markers for dendritic cells (B-D) and macrophage cells (E,F). 7-Aminoactinomycin D (7-AAD) and CD45 were used to exclude dead and non-hematopoietic cells, respectively. (TIF) [file pntd.0006417.s003.tif]

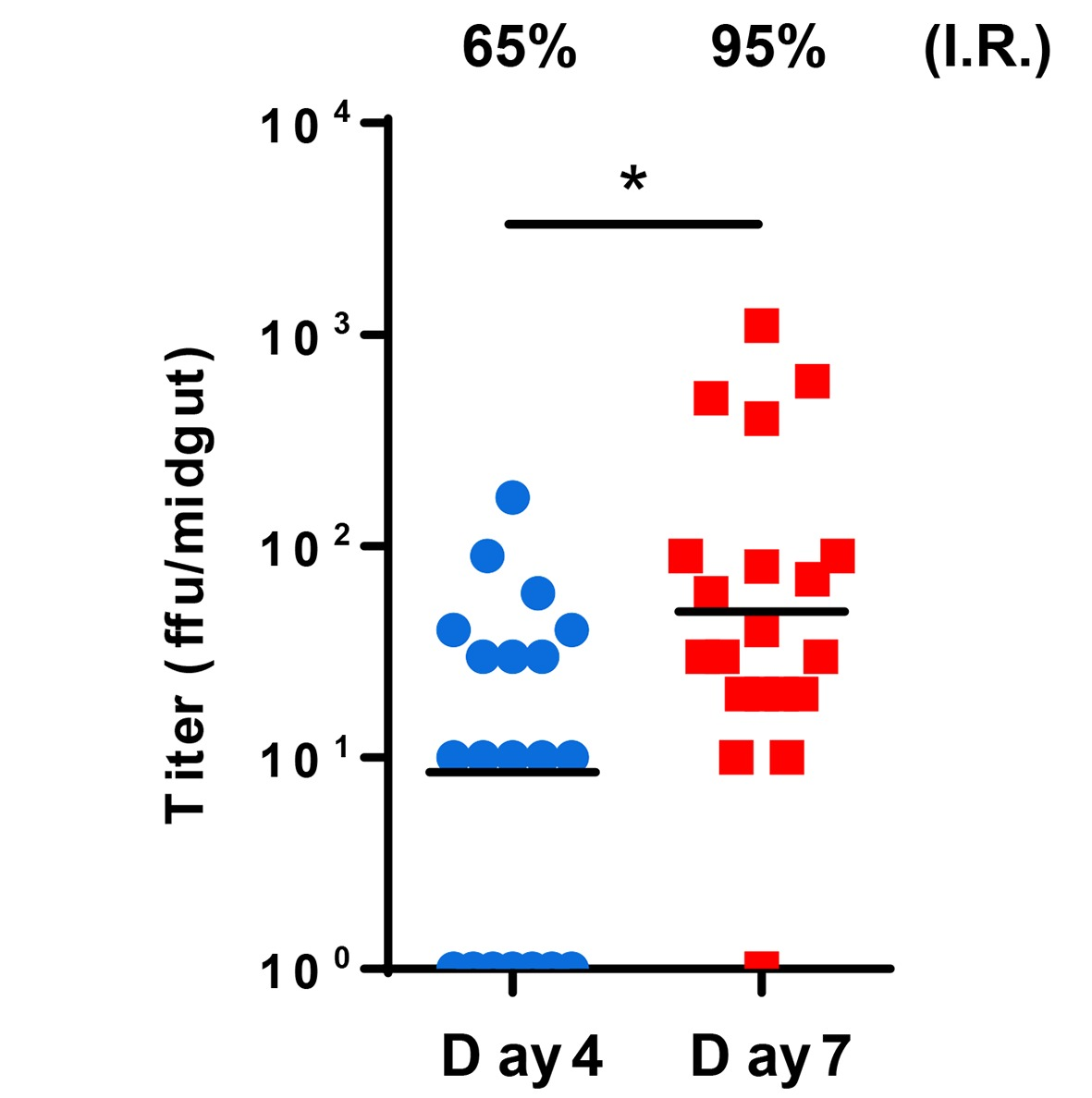

Supplement: S3 Fig — A. aegypti mosquitoes were injected in the thorax with ZIKV (400 pfu/mosquito) and viral titers were determined by focus-forming assay by homoginizing mosquito midgut 4 or 7 days later (n = 20).The ZIKV-infection rate (I.R.) was calculated. (TIF) [file pntd.0006417.s004.tif]

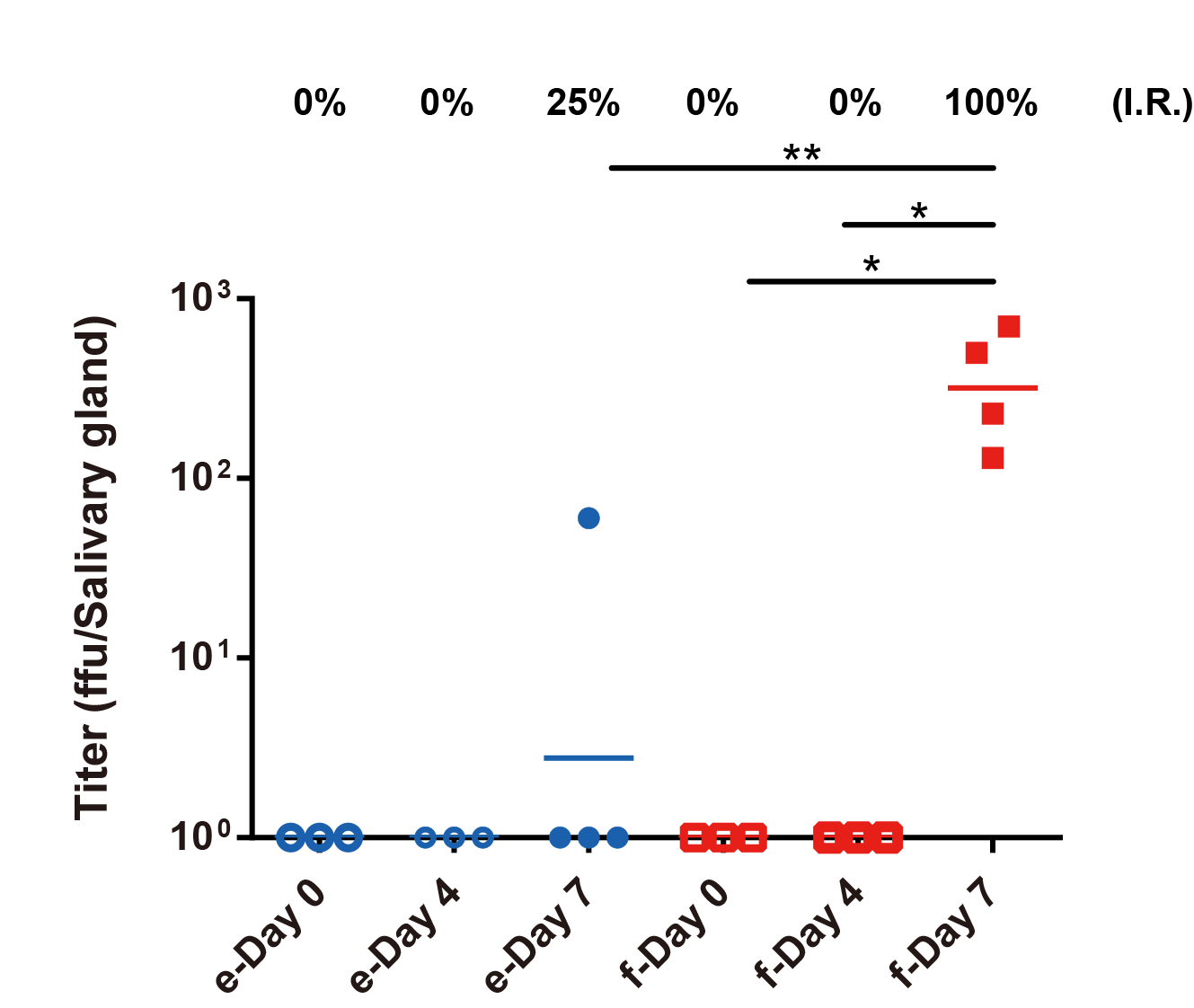

Supplement: S4 Fig — The salivary glands of the Group e and f mosquitoes which took blood meals from the ZIKV-infected mice (Group b and d mice, respectively, in Fig 5D; day 2 post-ZIKV infection) were isolated right after blood meal or on Day 4 or 7 post-blood meal and subject to virus titration by focus forming assay (n = 3–4). Infection rate was calculated. (TIF) [file pntd.0006417.s005.tif]

**Supplementary Table**

**Table S1.** The p,t and df values of the significant difference in Figures.


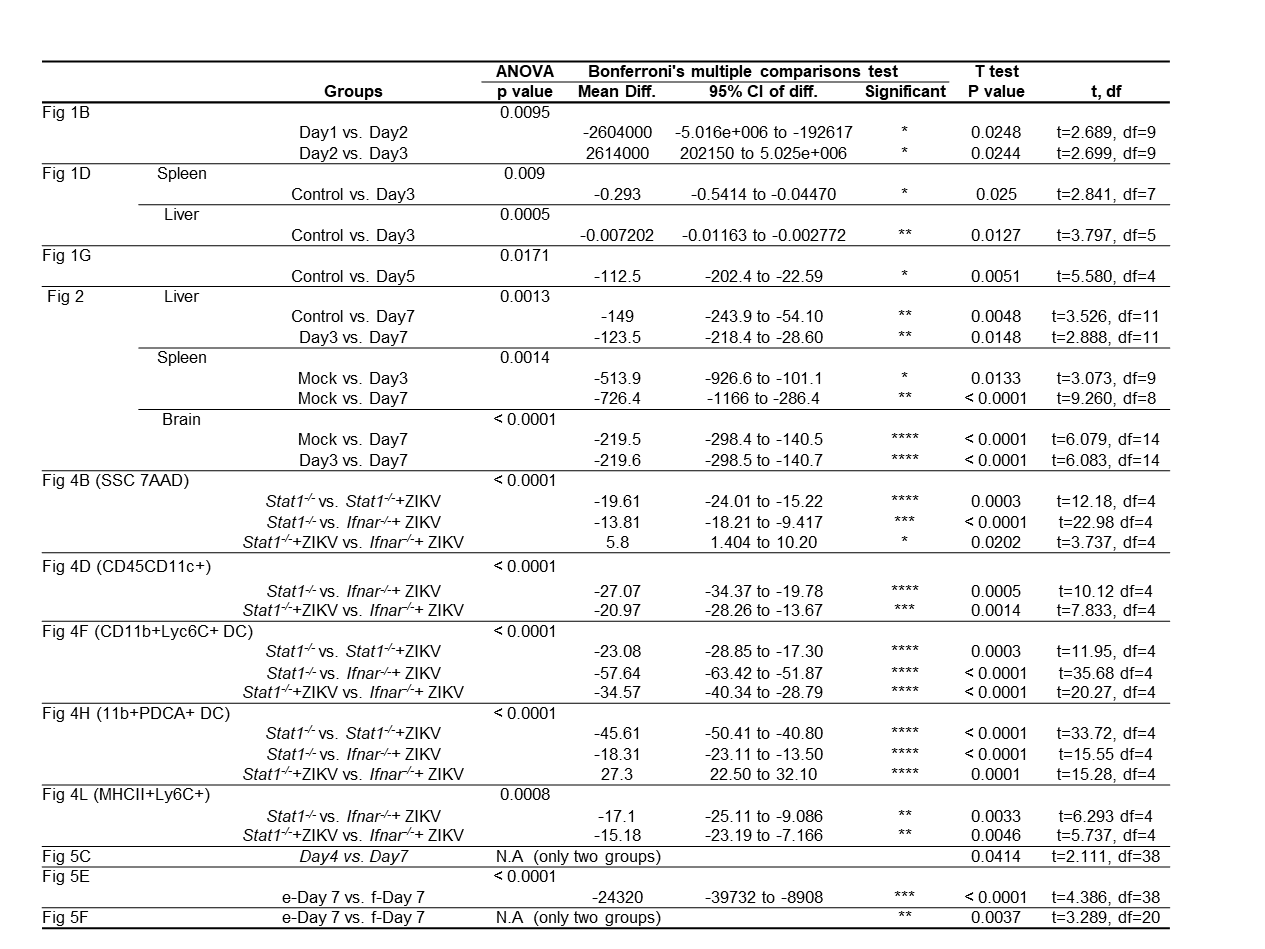

Supplement: S1 Table — (DOCX) [file pntd.0006417.s006.docx]
